# Augmentation of Extracellular ATP Synergizes With Chemotherapy in Triple Negative Breast Cancer

**DOI:** 10.3389/fonc.2022.855032

**Published:** 2022-04-20

**Authors:** Jasmine M. Manouchehri, Jharna Datta, Natalie Willingham, Robert Wesolowski, Daniel Stover, Ramesh K. Ganju, William E. Carson, Bhuvaneswari Ramaswamy, Mathew A. Cherian

**Affiliations:** Comprehensive Cancer Center, The Ohio State University, Columbus, OH, United States

**Keywords:** ATP, breast cancer, paclitaxel, P2RX4, P2RX7

## Abstract

**Introduction:**

Breast cancer affects two million patients worldwide every year and is the most common cause of cancer-related death among women. The triple-negative breast cancer (TNBC) sub-type is associated with an especially poor prognosis because currently available therapies fail to induce long-lasting responses. Therefore, there is an urgent need to develop novel therapies that result in durable responses. One universal characteristic of the tumor microenvironment is a markedly elevated concentration of extracellular adenosine triphosphate (eATP). Chemotherapy exposure results in further increases in eATP through its release into the extracellular space of cancer cells *via* P2RX channels. eATP is degraded by eATPases. Given that eATP is toxic to cancer cells, we hypothesized that augmenting the release of eATP through P2RX channels and inhibiting extracellular ATPases would sensitize TNBC cells to chemotherapy.

**Methods:**

TNBC cell lines MDA-MB 231, Hs 578t and MDA-MB 468 and non-tumorigenic immortal mammary epithelial MCF-10A cells were treated with increasing concentrations the chemotherapeutic agent paclitaxel in the presence of eATPases or specific antagonists of P2RXs with cell viability and eATP content being measured. Additionally, the mRNA, protein and cell surface expressions of the purinergic receptors P2RX4 and P2RX7 were evaluated in all examined cell lines *via* qRT-PCR, western blot, and flow cytometry analyses, respectively.

**Results:**

In the present study, we observed dose-dependent declines of cell viability and increases in eATP of paclitaxel-treated TNBC cell lines in the presence of inhibitors of eATPases, but not of the MCF-10A cell line. These effects were reversed by specific antagonists of P2RXs. Similar results, as those observed with eATPase inhibitors, were seen with P2RX activators. All examined cell lines expressed both P2RX4 and P2RX7 at the mRNA, protein and cell surface levels.

**Conclusion:**

These results reveal that eATP modulates the chemotherapeutic response in TNBC cell lines, which could be exploited to enhance the efficacy of chemotherapy regimens for TNBC.

## Introduction

Breast cancer affects millions of women every year. At 47.8 new cases and 13.6 deaths per 100000 per year, it has the highest global incidence rate and is the most common cause of cancer-related mortality among women in 2020 (1). Patients with triple-negative breast cancer (TNBC) have a markedly worse outcome in comparison to other breast cancer subtypes due to the aggressive and rapidly progressive nature of the disease and lack of specific targeted therapies (2–4). Hence, there is a critical need for more effective therapeutic strategies.

One universal characteristic of cancer is a marked elevation in extracellular adenosine triphosphate (eATP) (5–7). Under physiological conditions, the concentration of eATP is extremely low, in the 0-10 nanomolar (nM) range as compared to intracellular levels of 3 to 10 millimolar (mM), a difference of more than 10^6^-fold (8). However, eATP concentrations are markedly elevated in neoplastic and inflamed tissues, into the range of 100s of micromoles/liter (8).

Purinergic P2 receptors (P2Rs), integral plasma membrane receptors activated by ATP, are divided into the ionotropic P2 (P2RX) and metabotropic P2 (P2RY) sub-types (9). P2XRs, of which there are seven sub-types, are ATP-gated ion channels that are inducibly permeable to cations. With prolonged activation, P2RX7 channels become non-selectively permeable, resulting in the diffusion of high molecular weight molecules such as ATP; IL-1β and IL-18 release; large molecular weight dye uptake; K^+^ efflux; Na^+^ and Ca^2+^ influx; membrane phosphatidylserine-flip; membrane blebbing and cell death (10–14). Most P2RXs are activated by ATP concentrations in the nanomolar to low micromolar range, but P2RX7 activation requires millimolar concentrations of eATP (15–17). However, because P2RXs can homo and heterotrimerize to form functional channels with intermediate properties, ATP-dependent interactions between P2RX4 and the C-terminus of P2RX7 can potentiate P2RX7-dependent cell death (18).

Extracellular ATP release occurs through a variety of mechanisms, including tumor necrosis and apoptosis, vesicular exocytosis, active efflux *via* ATP-binding cassette subfamily C member 6 (ABCC6) and the ankylosis gene product ANK and diffusion *via* P2RX7 and Pannexin1 channels (10, 11, 19–24). Multiple pathways for eATP disposal have been described. These pathways hydrolyze nucleotides and limit their availability to activate nucleotide-specific P2Rs while increasing the concentration of extracellular nucleosides such as adenosine (25). There are four major classes of ecto-nucleotidases, including ecto-nucleoside triphosphate diphosphohydrolases (E-NTPDase), 5’ nucleotidases, ecto-nucleotide pyrophosphatases/phosphodiesterases (E-NPPase), and tissue non-specific alkaline phosphatases (TNAP) (25). E-NTPDases, which are nucleotide specific, are believed to be the major degradative enzymes for eATP. Extracellular 5’nucleotidase, which is classified as CD73 as per the cluster of differentiation system, catalyzes the conversion of AMP to adenosine and inorganic phosphate. Thus, eATP levels result from a balance between numerous synthetic and secretory pathways and degradative and endocytic pathways.

When cancer cells are exposed to cytotoxic chemotherapy, there is a release of ATP and K^+^ ions through P2RXs such as P2RX7 and P2RX4 into the extracellular space along with an influx of Ca^2+^ ions (17, 26–28). Exposure of various epithelial cancer cell lines to elevated eATP in culture and xenografts results in growth arrest or cell death (29–31). Notably, P2XR7 activation is a prerequisite for inflammasome activation, IL-1 and IL-18 secretion, and a highly inflammatory form of programmed cell death known as pyroptosis, which can lead to bystander cell death and immune activation (18). In addition, ATP has been administered to patients with advanced cancers with minimal side effects, and ATP administered in mice was associated with inhibitory effects on cancer cells (30, 32, 33).

Overall, these data suggest that the extracellular purinergic signaling pathway may be a promising target for cancer therapeutics. We hypothesized that increased eATP would increase the response to chemotherapy in TNBCs through the activation of P2RX channels, leading to increases in non-selective membrane permeability, the release of eATP and increased cancer cell death.

## Materials and Methods

### Cell Culture and Drugs and Chemicals

Breast cancer cell lines MDA-MB 231 (ATCC HTB-26, RRID : CVCL_0062), MDA-MB 468 (ATCC HTB-132, RRID : CVCL_0419), Hs 578t (ATCC HTB-126, RRID : CVCL_0332), and HEK-293T ATCC Cat# CRL-3216, RRID : CVCL_0063) were maintained in DMEM (Corning) and supplemented with 10% FBS (Cytiva), 1% MEM non-essential amino acids (Gibco), 1 mM sodium pyruvate (Gibco), 4 mM L-glutamine (Gibco) and antimicrobial agents (100 units/ml Penicillin, 100 µg/ml streptomycin, and 0.25 µg/ml amphotericin B) (Gibco). Non-tumorigenic immortal mammary epithelial MCF-10A cells (ATCC Cat# CRL-10317, RRID : CVCL_0598) were maintained in DMEM/F12 (Gibco) supplemented with 5% horse serum (Gibco), hydrocortisone (Sigma), epidermal growth factor (Sigma), cholera toxin (Sigma), insulin (Sigma) and antimicrobial agents. All cell lines were authenticated and were maintained in a humidified incubator at 37°C and 5% CO_2_.

The following drugs and chemicals were used: dimethyl sulfoxide/DMSO (Sigma), ATP (Sigma), UTP (Sigma), paclitaxel (Calbiochem), sodium polyoxotungstate or POM-1 (Tocris), PSB 069 (Tocris), levamisole hydrochloride (Abcam), A438079 (Tocris), 5-BDBD (Tocris), ENPP1 inhibitor C (Cayman Chemical, Ann Arbor, MI), SBI-425 (MedChemExpress), etidronate disodium and ivermectin (Sigma). ATP and POM-1 were dissolved in nuclease-free water (Invitrogen); paclitaxel, A438079, Iso-PPADS, 5-BDBD, SBI-425, ENPP1 inhibitor C, levamisole hydrochloride, etidronate disodium (Sigma), and ivermectin were dissolved in DMSO. **Table 1** shows drugs’ concentrations and functions; we optimized the drug concentrations that were applied for the different assays by using previously described drugs’ concentrations as starting points (34–43). Drugs were added to media at designated concentrations and applied to cells in an *in vitro* system.

**Table 1:** Drug concentrations and functions. The table shows the drugs administered, their concentrations applied and their functions.

Drug	Concentration(s)	Function
Paclitaxel	12.5, 25, 50, 100 μmol/L	Chemotherapeutic agent
Iso-PPADs	20 μmol/L	P2RX inhibitor
A437809	20 μmol/L	P2RX7 inhibitor
5-BDBD	20 μmol/L	P2RX4 inhibitor
POM-1	10 μmol/L	ecto-nucleoside triphosphate diphosphohydrolases (ENTPDase) inhibitor
PSB 069	10 μmol/L	ENTPDase inhibitor
ENNP1 C inhibitor	10 μmol/L	ecto-nucleoside pyrophosphatases/ phosphodiesterase 1 (ENPP1) inhibitor
SBI-425	10 μmol/L	tissue-nonspecific alkaline phosphatase (TNAP) inhibitor
Levamisole hydrochloride	10 μmol/L	TNAP inhibitor
Etidronate disodium	10 μmol/L	protein tyrosine phosphatase inhibitor
Ivermectin	20 μmol/L	P2RX4 and P2RX7 activator

### Cell Viability and eATP Assays

**Figure 1:**
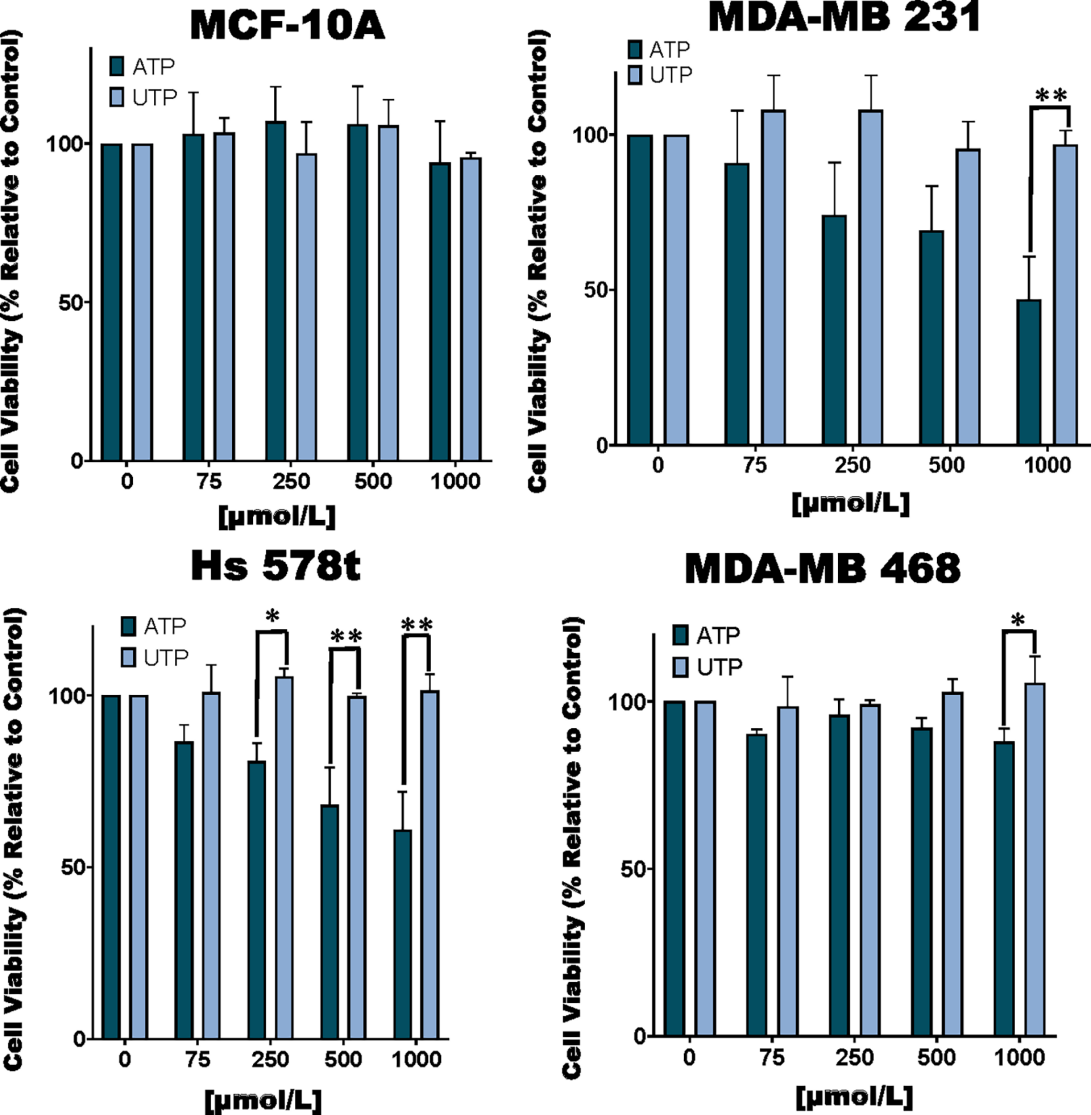
Cell viability of ATP and UTP-treated cells. TNBC MDA-MB 231, Hs 578t and MDA-MB 468 cell lines and non-tumorigenic immortal mammary epithelial MCF-10A cells were treated for 48 hours with increasing concentrations of ATP or UTP, and cell viability was measured with the PrestoBlue HS assay. Error bars represent standard deviations calculated from three independent experiments performed in triplicate. The student’s t-test was applied to the to ascertain significance. * represents p < 0.05 and ** represents p < 0.01 when comparing ATP to UTP.

**Figure 2:**
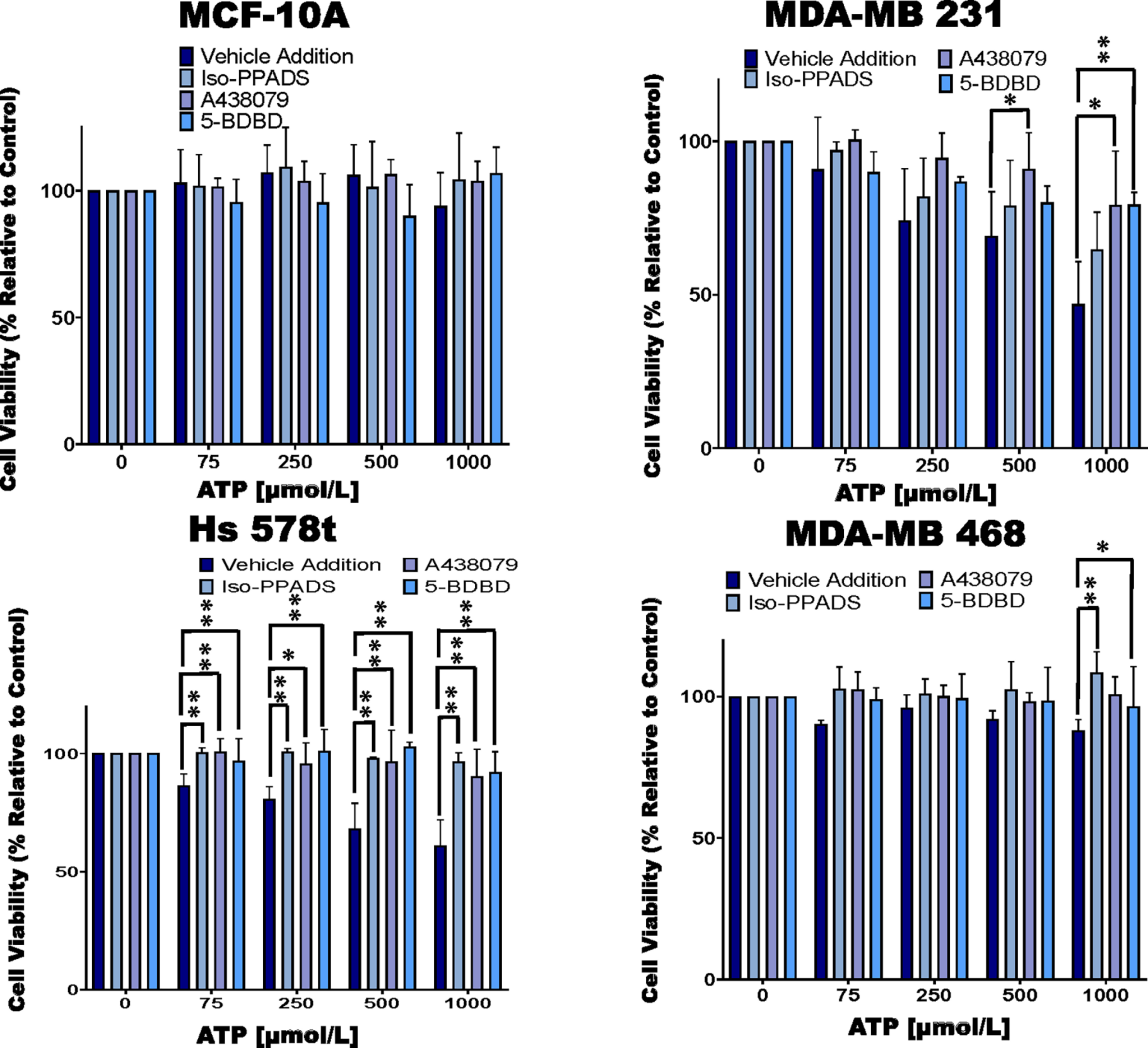
Cell viability of ATP-treated cells in the presence of P2RX inhibitors. TNBC cell lines and MCF-10A cells were treated for 48 hours with increasing concentrations of ATP in the presence of the P2RX inhibitor Iso-PPADS (20 µmol/L), the P2RX7 inhibitor A438079 (20 µmol/L) or the P2RX4 inhibitor 5-BDBD (20 µmol/L) or vehicle addition, and cell viability was measured using the PrestoBlue HS assay. Error bars represent standard deviations calculated from three independent experiments performed in triplicate. One way ANOVA with Tukey’s HSD was applied to ascertain significance. * represents p < 0.05 and ** represents p < 0.01 when comparing vehicle addition to Iso-PPADS, A438079 or 5-BDBD.

**Figure 3:**
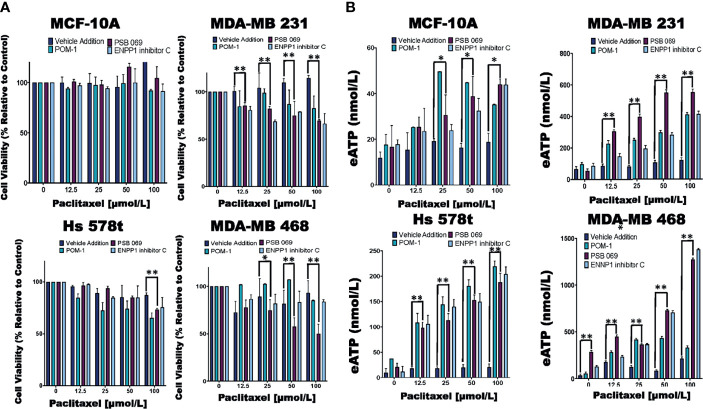
Comparing eATP release from paclitaxel-treated cells in the presence of inhibitors or vehicle addition. **(A)** TNBC and MCF-10A cells were treated with increasing concentrations of paclitaxel and the nucleoside phosphohydrolase inhibitors POM-1 (E-NTPDase inhibitor, 10 µmol/L), PSB 069 (E-NTPDase inhibitor, 10 µmol/L), ENNP1 inhibitor C (ENPP1 inhibitor, 10 µmol/L) or vehicle addition for six hours, and cell viability was measured using the PrestoBlue HS assay. Standard deviation was calculated from three independent experiments performed in triplicate. **(B)** eATP concentrations were measured in the supernatants of TNBC and MCF-10A cells after six hours of treatment with increasing concentrations of paclitaxel and nucleoside phosphohydrolase inhibitors or vehicle addition. Standard deviation was calculated from three independent experiments performed in triplicate. One way ANOVA with Tukey’s HSD was applied to ascertain significance. * represents p < 0.05 and ** represents p < 0.01 when comparing vehicle addition to PSB 069. We highlighted just the significance in the presence of PSB 069 because the cell viability results were consistently significant.

**Figure 4:**
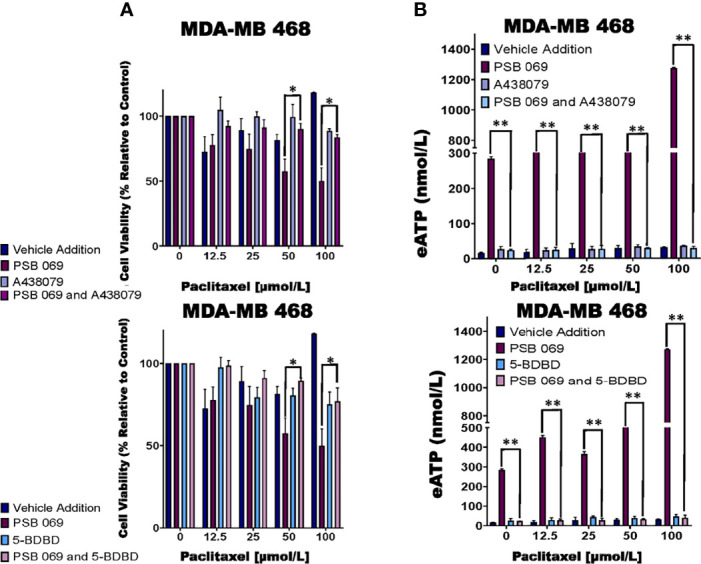
Examining the influence of P2RX inhibitors in combination with E-NTPDase inhibitor on cell viability and eATP release in paclitaxel-treated cells. **(A)** Paclitaxel-treated breast cancer MDA-MB 468 cell lines were treated for six hours with P2RX7 inhibitor A438079 (20 µmol/L) or P2RX4 inhibitor 5-BDBD (20 µmol/L) in the presence or absence of PSB 069 (10 µmol/L), and cell viability was measured by applying PrestoBlue HS assay. Standard deviation was calculated from three independent experiments performed in triplicate. We used the same values for both graphs for vehicle addition and PSB 069. **(B)** eATP concentrations were measured in the supernatants of paclitaxel-treated MDA-MB 468 cells after six hours of treatment. Standard deviation was calculated from three independent experiments performed in triplicate. We used the to the same values for both graphs for vehicle addition and PSB 069. The student’s t-test was applied to the applicable assays to ascertain significance. * represents p < 0.05 and ** represents p < 0.01 for A438079 and PSB 069 or 5-BDBD and PSB 069 when compared to PSB 069 alone.

**Figure 5:**
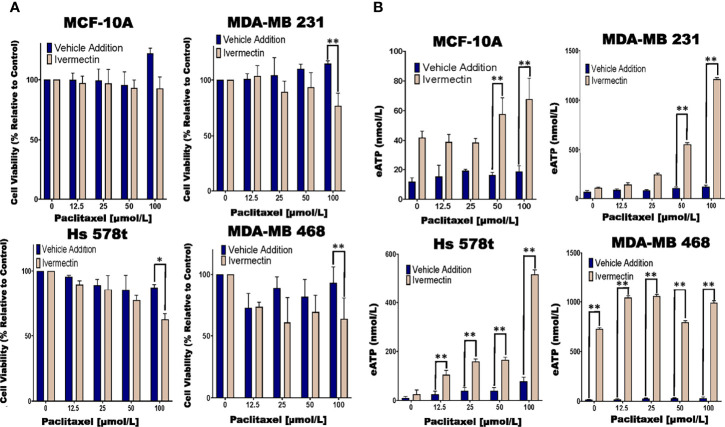
Determining relative eATP content and cell viability in paclitaxel-treated cells in the presence of ivermectin or vehicle addition. **(A)** The graphs represent cell viability as measured using the Presto Blue HS assay +/- standard deviation from three independent experiments performed in triplicate in TNBC and MCF-10A cells after six hours of treatment with increasing concentrations of paclitaxel and the P2RX4 and P2RX7 activator ivermectin (10 µmol/L) or vehicle addition. **(B)** eATP content was measured in the supernatants of paclitaxel-treated TNBC and MCF-10A cell lines in the presence of the P2RX4 and P2RX7 activator ivermectin (10 µmol/L) or vehicle addition. The student’s t-test was applied to the applicable assays to ascertain significance. * represents p < 0.05 and ** represents p < 0.01 when comparing ivermectin to vehicle addition.

TNBC cell lines, MDA-MB 231, Hs 578t, MDA-MB 468 cells and non-tumorigenic immortal mammary epithelial MCF-10A cells were plated on 96 well plates (Costar) at a high density of 25,000 cells/well and after 24 hours treated with paclitaxel, inhibitors, or ATP for 6 or 48 hours. PrestoBlue™ HS cell viability reagent (Invitrogen) was added to each well according to the manufacturer’s instructions. Fluorescence readings (excitation and emission ranges: 540–570 nm and 580–610 nm) were obtained using a Bioteck Synergy HT plate reader. ATP was measured in supernatants according to the protocol described by the ATPlite 1 step Luminescence Assay System (PerkinElmer). Luminescence readings were measured on a Bioteck Synergy HT plate reader. The student’s t-test was applied to the applicable assays (**Figures 1**, **4**, **5**) to ascertain significance. * represents p<0.05 and ** represents p<0.01; for **Figure 1** comparing ATP to UTP, for **Figure 4** comparing PSB alone to PSB 069 and A438079 or to PSB 069 and 5-BDBD, for **Figure 5** comparing vehicle addition (paclitaxel alone) and ivermectin. For **Figures 2**, **3**, one way ANOVA with Tukey’s HSD (Honestly Significant Difference) was applied to ascertain significance. For **Figure 2** * represents p<0.05 and ** represents p<0.01 when comparing vehicle addition to Iso-PPADS, A438079 or 5-BDBD. For **Figure 3** * represents p<0.05 and ** represents p<0.01 when comparing vehicle addition to PSB 069. We highlighted just the significance in the presence of PSB 069 because the cell viability results were consistently significant.

### RNA Analysis of P2RX4 and P2RX7

MDA-MB 231, Hs 578t, MDA-MB 468, cell lines and MCF-10A cells were maintained under standard conditions in subconfluent cultures. RNA was extracted *via* the TRIzol method (*Invitro*gen), and qRT-PCR was performed on a Bio-Rad T100 thermal cycler using the following exon-exon junction-spanning primers: for P2RX4, the forward primer TGGCGGATTATGTGATACCAGC and the reverse primer GTCGCATCTGGAATCTCGGG; for P2RX7, the forward primer GTGTCCCGAGTATCCCACC and the reverse primer GGCACTGTTCAAGAGAGCAG; and for GAPDH forward primer GTCGTATTGGGCGCCTGGTC and the reverse primer TTTGGAGGGATCTCGCTCCT. The student’s t-test was applied to the applicable assays to ascertain significance. * represents p<0.05 and ** represents p<0.01 relative to MCF-10A; + represents p<0.05 and ++ represents p<0.01 relative to HEK293-empty vector transfected.

### Western Blot Analysis of P2RX4 and P2RX7

**Figure 6:**
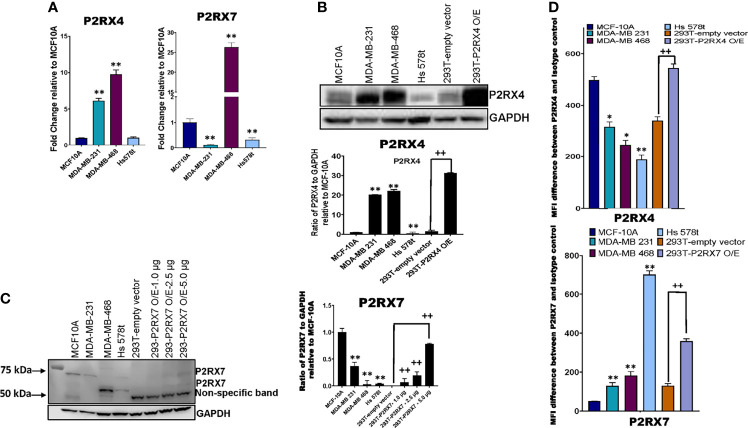
mRNA and protein expression analysis of P2RX4 and P2RX7 for all cell lines. **(A)** qRT-PCR was performed on mRNA of TNBC cell lines and MCF-10A cells using specific primers for P2RX4 and P2RX7. * represents p < 0.05 and ** represents p < 0.01. TNBC cell lines, MCF-10A cells, and HEK 293T cells transfected with P2RX4 or P2RX7 as positive controls were probed for **(B)** P2RX4 and **(C)** P2RX7, and GADPH was used as a loading control for western blot analysis repeated twice. HEK 293T cells transfected with P2RX7 were loaded at increasing protein concentrations of 1.0 µg, 2.5 µg, and 5.0 µg combined with lysates of control vector-transfected cells to keep the total loaded protein the same in each lane. Densitometry analysis was performed using Image Studio on the 75 kDa P2RX7 band. The student’s t-test was applied to the applicable assays to ascertain significance. * represents p<0.05 and ** represents p < 0.01 relative to MCF-10A; + represents p < 0.05 and ++ represents p < 0.01 relative to HEK293-empty vector transfected. **(D)** The calculated difference in mean fluorescence intensity (MFI) values between TNBC cell lines, MCF-10A cells, and HEK 293T cells transfected with P2RX4 or P2RX7 as positive controls stained with P2RX4 or P2RX7 specific antibody and the isotype control for the different cell lines examined. * represents p < 0.05 and ** represents p < 0.01 relative to MFI difference in MCF-10A cells; + represents p < 0.05 and ++ represents p < 0.01 relative to MFI difference in HEK293-empty vector transfected. O/E represents overexpressed.

Total cell lysates were prepared in lysis buffer (50 mM Tris HCl at pH 8.0, 1.0 mM EDTA, 1% SDS, and 1% Igepal CA630) with a protease inhibitor cocktail (Thermo Scientific). The lysates were sonicated, placed on ice for 30 minutes, and spun at 10,000 rpm for 10 minutes at 4°C to collect the cleared supernatants for analysis. Protein quantification was performed using the Pierce BCA Protein Assay (Thermo Scientific) and absorbance readings taken at 595 nm. Protein samples (P2RX4: 100 µg, positive control-293T/empty vector 99.5 µg + 293T/O/E P2RX4 0.5 µg and P2RX7: 200 µg, positive control-293T/empty vector 199, 197.5, or 195 µg + 293T/O/E P2RX7 1, 2.5 or 5 µg, respectively, as shown in **Figure 6**) were denatured with 4X Laemmli sample buffer (250 mM Tris-HCl, 8% SDS, 40% glycerol, 8% BME, and 0.06% Bromophenol Blue) at 98°C for 5 minutes and separated on 12% sodium dodecyl sulfate (SDS)-polyacrylamide gels (Invitrogen). Proteins were transferred to nitrocellulose membranes (Millipore) employing the wet transfer method (Bio-Rad, Hercules, CA). The membranes were blocked with TBS-T (0.15 M NaCl, 0.02 M Tris-HCl, pH 7.4 and 0.1% Tween-20) containing non-fat milk at room temperature for an hour and incubated overnight at 4°C with a primary antibody: P2RX4 (1:500 dilution; Cell Signaling Technology, Cat# 70659, RRID : AB_2799789) and anti-P2RX7 (1:200 dilution; Cell Signaling Technology, Cat# 13809, RRID : AB_2798319), diluted in 5% BSA (GoldBio) or 5% non-fat milk. The membranes were washed in TBS-T (0.15 M NaCl, 0.02 M Tris HCl, pH 7.4), incubated with horseradish peroxidase (HRP)-conjugated secondary anti-rabbit/mouse antibodies diluted in 5% non-fat milk (1:5000) for one hour and washed in TBS-T. The blots were analyzed using enhanced chemiluminescence Immobilon Western Chemiluminescent HRP Substrate (Millipore). The membranes were then stripped and re-probed for GAPDH (Cell Signaling Technology) as the internal loading control. Densitometry was performed on Image Studio (Licor). The student’s t-test was applied to the applicable assays to ascertain significance. * represents p<0.05 and ** represents p<0.01 relative to MCF-10A; + represents p<0.05 and ++ represents p<0.01 relative to HEK293-empty vector transfected.

### Flow Cytometry Analysis of P2RX4 and P2RX7

MDA-MB 231, MDA-MB 468, Hs 578t, MCF-10A cells, and HEK 293T cells were maintained as previously described. HEK 293T were transfected with either P2RX4 or P2RX7 expression plasmids derived from pcDNA3.1 (RRID: Addgene_79663) using Lipofectamine 3000 (Thermo Fisher Scientific). Cells were detached with accutase (Thermo Fisher Scientific). One million cells were washed in PBS with 0.05% BSA, stained with P2RX7 –FITC (Sigma Aldrich, # P8997, RRID : AB_477416) antibodies or stained with rabbit IgG Isotype Control-FITC (Invitrogen, Cat# PA5-23092, RRID : AB_2540619), or with anti-P2RX4 (Cell Signaling Technology) plus goat anti-rabbit IgG (H+L) secondary antibody-FITC (Novus Biologicals, # NB 7168, RRID : AB_524413) or IgG isotype control plus secondary antibody-FITC in Flow Cytometry Staining Buffer (2% FBS, 0.02% sodium azide and PBS). Analysis was performed on BD FACS Fortessa using the FITC channel (530/30 nm) and Flowjo software (RRID: SCR_008520). The student’s t-test was applied to the applicable assays to ascertain significance. * represents p<0.05 and ** represents p<0.01 relative to MFI difference in MCF-10A cells; + represents p<0.05 and ++ represents p<0.01 relative to MFI difference in HEK293-empty vector transfected. O/E represents overexpressed.

## Results

### Cell Viability of ATP and UTP-Treated Cells and the Impact of P2RX Inhibitors on Cell Viability of ATP-Treated Cells

We examined the toxic effects of ATP using UTP as a control. We observed a dose-dependent loss of viability in TNBC MDA-MB 231, MDA-MB 468 and Hs 578t cell lines upon exposure to ATP but not UTP and not in non-tumorigenic immortal mammary epithelial MCF-10A cells (**Figure 1**). For example, in MDA-MB 231 cells treated with increasing concentrations of ATP, there was a mean loss of viability between 10 and 50%. Similar effects were observed in ATP-treated Hs 578t cells with a mean loss in viability between 15 and 40%. For ATP-treated MDA-MB 468 cells, there was a mean loss of viability between 10 and 13%. We did not see any significant change in cell viability in ATP-treated MCF-10A cells. There were no significant changes in viability in UTP-treated cells.

We next treated the cells with various purinergic receptor antagonists to determine whether P2RX receptors mediate the effects of eATP on cell viability (**Figure 2**). As with **Figure 1**, we did not see any change in cell viability in ATP-treated MCF-10A cells and therefore, did not see any additional changes with exposure to P2RX antagonists. However, for ATP-treated TNBC cells, in the absence of inhibitors we saw decreases in viability that were attenuated by P2RX inhibitors, which decreased their sensitivity to inhibition by ATP. For example, in MDA-MB 231 cells treated with increasing concentrations of ATP, when compared to vehicle addition, there was an improvement in cell viability between 7 and 15% when exposed to the non-specific P2RX inhibitor Iso-PPADS (20 µmol/L), an improvement in cell viability between 10 and 30% when exposed to the P2RX7 inhibitor A438079 (20 µmol/L), and an improvement in cell viability between 0 and 30% when exposed to the P2RX4 inhibitor 5-BDBD (20 µmol/L). Similar effects were seen in ATP-treated Hs 578t cells when compared to vehicle addition, there was an improvement in cell viability between 14 and 33% when exposed to Iso-PPADS, an improvement in cell viability between 14 and 30% when exposed to A438079, and an improvement in cell viability between 10 and 32% when exposed to 5-BDBD. For ATP-treated MDA-MB 468 cells as compared to vehicle-addition, there was an improvement in cell viability between 12 and 23% when exposed to Iso-PPADS, an improvement in cell viability between 10 and 12% when exposed to A438079, but no significant improvement in cell viability when exposed to 5-BDBD.

### Examining the Effects of eATPase Inhibitors on Cell Viability and eATP Release

We next studied the effects of combinations of eATPase inhibitors with chemotherapy (paclitaxel) to determine their effects on the efficacy of chemotherapy. For these experiments, all the cell lines were treated for six hours to simulate the duration of systemic exposure in patients (**Figure 3**). For this reason, we did not see changes in the viability of cells treated with paclitaxel alone. Additionally, there were decreases in cell viability in the paclitaxel-treated TNBC cell lines in the presence of POM-1 and the ENPPase inhibitor ENPP1 inhibitor C, but these results were not consistently significant. For MDA-MB 231 cells treated with increasing concentrations of paclitaxel, there was a mean decrease in cell viability between 15 to 30% in the presence of the E-NTPDase inhibitor PSB 069 when compared to vehicle addition. Similarly for paclitaxel-treated Hs 578t cells in in the presence of PSB 069, there was a loss of viability between 0 to 14%. For paclitaxel-treated MDA-MB 468 cells there was a decrease in cell viability between 0 to 50% in the presence of PSB 069 as compared to vehicle addition. However, there was no significant change in the viability of paclitaxel-treated MCF-10A cells in the presence of the three inhibitors (**Figure 3A**). We also confirmed that under these experimental conditions, treatment with the eATPase inhibitors alone did not significantly change cell viability in any of the cell lines (**Supplementary Figure 1**). Therefore, PSB 069 most potently and consistently decreased the viability of TNBC cell lines when combined with paclitaxel.

In the same experiments, we measured the amount of eATP in the supernatants of chemotherapy-treated cells (**Figure 3B**). Treating cells with paclitaxel alone produced quite modest increases in eATP that were generally not statistically significant when compared to vehicle control: for MDA-MB 231 cells treated with increasing concentrations of paclitaxel, eATP increments ranged from 66 to 120 nmol/L, for Hs 578t, from 9 to 20 nmol/L, for MDA-MB 468, eATP increased from 34 to 213 nmol/L, and for MCF-10A, to 12 to 18 nmol/L. However, in the presence of inhibitors, we saw significant increases in eATP levels. For instance, in MDA-MB 231 cells treated with increasing concentrations of paclitaxel, the increments in eATP concentration upon treatment with POM-1 ranged from 130 to 450 nmol/L), with PSB 069 54 to 550 nmol/L and with ENPP1 inhibitor C 86 to 410 nmol/L. Similarly, for Hs 578t cells treated with increasing concentrations of paclitaxel, with POM-1 the increase in eATP ranged from 34 to 219 nmol/L, with PSB 069 21 to 188 nmol/L and with ENPP1 inhibitor C from 12 to 204 nmol/L. For MDA-MB 231 cells treated with increasing concentrations of paclitaxel, the increments in eATP concentration with POM-1 ranged from 84 to 450 nmol/L, with PSB 069 between 284 nmol/L to 1.3 µmol/L and with ENPP1 inhibitor C 129 nmol/L to 1.4 µmol/L. For MCF-10A cells treated with increasing concentrations of paclitaxel, the increments in eATP concentration increased with POM-1 ranged from 18 to 40 nmol/L, with PSB 069 16 to 41 nmol/L and with ENPP1 inhibitor C 18 to 39 nmol/L. Thus, ENTPDase and NPPase inhibitors significantly increased eATP release upon chemotherapy treatment although the magnitude of this increase was much higher in TNBC cell lines than in immortal mammary epithelial cells.

### Examining the Effects of P2RX Inhibitors on the E-NTPDase Inhibitor-Induced Exaggerated Loss of Cell Viability and eATP Release

Of the eATPase inhibitors tested, we consistently saw an exaggerated loss of cell viability with the E-NTPDase inhibitor PSB 069. Therefore, we sought to determine if the increased loss of cell viability in the presence of PSB 069 is dependent on eATP induced activation of P2RX4 or P2RX7 (**Figure 2**). We chose the MDA-MB 468 cell line because the baseline effects of PSB 069 were maximal and therefore, the reversal of these effects would be most meaningful.

We did see reversal of the effects of PSB 069 on cell viability and eATP release upon concurrent treatment with both the P2RX7 inhibitor A438079 and the P2RX4 inhibitor 5-BDBD (**Figure 4A**). In paclitaxel - treated MDA-MB 468 cells, there was an improvement in viability that ranged from 8 to 34% for the combination of A438079 with PSB 069 when compared to vehicle addition with PSB 069 and an improvement in cell viability ranging from 24 to 27% in the presence of 5-BDBD and PSB 069 when compared to vehicle addition with PSB 069. These results show that the increased loss of cell viability observed when PSB 069 is combined with paclitaxel is dependent on the activation of P2RX4 and P2RX7 by eATP.

In the same experiments, we determined the effects of A438079 and 5-BDBD on eATP release in MDA-MB 468 cells treated with 10 µmol/L PSB 069 and increasing concentrations of paclitaxel **(** **Figure 4B****)**. There was a decrease in eATP from a range of between 284 nmol/L and 1.3 µmol/L to a range of between 40 and 70 nmol/L when A438079 was combined with PSB 069. There was a decrease in eATP from a range of between 284 nmol/L and 1.3 µmol/L to a range between 30 to 80 nmol/L when 5-BDBD was combined with PSB 069. These results show that the increased eATP release observed when PSB 069 is combined with paclitaxel is dependent on the activation of P2RX4 and P2RX7 by eATP.

Previous reports indicate that tissue non-specific alkaline phosphatase also metabolizes eATP. We sought to ascertain if two tissue non-specific alkaline phosphatase inhibitors (SBI 425 and levamisole hydrochloride) could augment the effects on cell viability and eATP release in paclitaxel-treated cells while using a protein tyrosine phosphatase inhibitor, etidronate disodium, as a control. Although we observed substantial changes in eATP upon treatment of the TNBC cell lines, there was no significant change in cell viability **(** **Supplementary Figure 2****)**.

### Evaluating the Impact of a P2RX Activator on Cell Viability and ATP Release in Chemotherapy-Treated Cells

Previous research had shown that ivermectin is a P2RX4 and P2RX7 activator (44, 45). Hence, we examined the effects of ivermectin on eATP and cell viability in chemotherapy-treated MCF10A cells and TNBC cell lines. We observed significant decreases in cell viability in paclitaxel and ivermectin-treated TNBC cell lines but not in MCF-10A cells (**Figure 5A**). As an example, for ivermectin addition (20 μmol/L) compared with vehicle addition to cells treated with increasing concentrations of paclitaxel, MDA-MB 231, Hs 578t and MDA-MB 468 cells showed between 3 to 35%, 7 to 38% and 6 to 50% mean decreases in cell viability, respectively.

In the same experiments, we also looked at eATP release upon exposure to the combined treatment of ivermectin and paclitaxel. For paclitaxel-treated cells, there were increases in eATP release in the presence of ivermectin when compared to the vehicle addition. These increases were much more dramatic in the TNBC cell lines than immortal mammary epithelial cells (**Figure 5B**). As an example, for MDA-MB 231 cells in the presence of ivermectin, eATP increased from a range of between 66 and 120 nmol/L (vehicle addition) to a range of between 108 nmol/L and 1.2 µmol/L, for Hs 578t cells eATP increased from a range of between 9 and 20 nmol/L (vehicle addition) to a range of between 25 and 517 nmol/L and for MDA-MB 468 cells eATP increased from a range of between 34 and 213 nmol/L (vehicle addition) to a range of between 730 nmol/L and 1 µmol/L. For MCF-10A cells in the presence of ivermectin, eATP increased from a range of between 12 and 18 nmol/L (vehicle addition) to a range of between 42 and 68 nmol/L. Therefore, ivermectin potentiated the effects of paclitaxel on TNBC cell lines.

### Expression of P2RX4 and P2RX7 in TNBC Cell Lines

We next sought to assess the expression of P2RX4 and P2RX7 mRNA and protein. qRT-PCR was performed on TNBC and MCF-10A cells with specific exon-exon junction-spanning primers for *P2RX4*, *P2RX7* and *GAPDH*, and fold change was calculated relative to the expression of the receptors in MCF-10A cells (**Figure 6A**). Some TNBC cell lines expressed more *P2RX4* mRNA in comparison to MCF-10A cells: MDA-MB 231 (5-fold; p=0.0012 and MDA-MB 468 (10-fold; p=0.0001); whereas Hs 578t cells expressed levels that were not significantly different (p>0.05). MDA-MB 231 and Hs 578t cells expressed significantly less *P2RX7* mRNA when compared to MCF-10A cells (p=0.0001 for both); whereas, MDA-MB 468 cells expressed 25-fold more *P2RX7* mRNA when compared to MCF-10A cells (p=0.0006).

Western blot analysis was performed on TNBC cell lines, MCF-10A cells and HEK 293T cells transfected with either a P2RX4 or P2RX7 expression plasmid as positive controls, probing for P2RX4 and P2RX7 with GAPDH as the internal loading control (**Figure 6B**). Two of three TNBC cell lines expressed more P2RX4 protein when compared to MCF-10A cells when assessed by semi-quantitative densitometry: MDA-MB 231 (20-fold; p=0.001), MDA-MB 468 (22-fold; p=0.001) while Hs 578t cells expressed significantly less P2RX4 protein (p=0.01). We separately probed for P2RX7 protein in the same cell lines. We did detect specific bands corresponding to the full-length glycosylated P2RX7A isoform (75kDa) in the MCF-10A cells but significantly less protein was detected in MDA-MB 231 and Hs 578t cells while a specific 69 kDa band was detected in MDA-MB 468 and Hs 578t cells; we did detect specific bands at 69 and 75 kDa in transfected 293T cells that increased in intensity with increasing loaded mass of lysate from P2RX7-transfected 293T cells. The P2RX7 expression plasmid incorporates the cDNA for the full-length P2RX7A isoform. The P2RX7 protein includes 5 N-linked glycosylation sites. Thus, the 69 kDa band corresponds to the unglycosylated form of the protein and the 75 kDa band likely represents the fully glycosylated form of the protein.

Given that unglycosylated form may not represent the plasma membrane-localized fraction of a protein, we used flow cytometry to quantitate the expression of P2RX7 and P2RX4 at the cell surface. Flow cytometry analysis was performed on TNBC, MCF-10A, and HEK 293T cells transfected with either vector control or P2RX4 expression plasmid as a positive control, probing for cell surface expression P2RX4 (**Figure 6D**) using a primary antibody that targets the extracellular domains of P2RX4. MDA-MB 231, Hs 578t and MDA-MB 468 expressed significantly less cell surface P2RX4 in comparison to MCF-10A cells. The calculated mean fluorescence intensity (MFI) difference between the P2RX4 specific and isotype control antibody for MCF-10A cells was 498, for MDA-MB 231 cells 316 (p=0.05 when compared to MFI difference for MCF-10A), for MDA-MB 468 cells 246 (p=0.02), and Hs 578t cells 189 (p=0.01).

We performed flow cytometry analysis on TNBC, MCF-10A, and HEK 293T cells transfected with either empty vector or P2RX7 expression plasmid as a positive control, probing for cell surface expression P2RX7 (**Figure 6D**) using a primary antibody that targets the extracellular domains of P2RX7. MDA-MB 231, Hs 578t and MDA-MB 468 expressed significantly more cell surface P2RX7 in comparison to MCF-10A cells. The calculated mean fluorescence intensity (MFI) difference between P2RX7 and the isotype control for MCF-10A cells was 51, for MDA-MB 231 cells 129 (p=0.003 when compared to MFI difference to MCF-10A), for MDA-MB 468 cells 182 (p=0.002), and for Hs 578t cells 703 (p=0.0001). Thus, all cell lines expressed both receptors at the cell surface when measured by flow cytometry, and all the TNBC cell lines expressed significantly more P2RX7 protein at the cell surface than immortal mammary epithelial cells.

Additionally, all cell lines were treated with 100 µmol/L paclitaxel and the cell surface expressions of P2RX4 and P2RX7 were examined with the calculated difference in MFI values between cells stained with P2RX4 or P2RX7 specific antibodies to cells stained with the corresponding isotype control (**Supplementary Figure 4**). Upon treatment, cell surface expression of P2RX7 increased significantly in some TNBC cell lines but this was not a consistent effect.

## Discussion

Chemotherapy by itself fails to ablate metastatic TNBC. Extracellular ATP, in the high micromolar to millimolar range, induces cytotoxicity in cancer cell lines. Chemotherapy is known to induce increases in eATP. We hypothesized that interventions that augment chemotherapy-induced increases in eATP would increase cancer cell death.

Our results show that inhibitors of E-NTPDases, ENPPases and TNAP all significantly increased the release of eATP with chemotherapy exposure. However, only the E-NTPDase inhibitor PSB 069, a sulfonated tetracyclic compound, but not POM-1, another E-NTPDase inhibitor, consistently and significantly increased chemotherapy-induced cell death. Both are inhibitors of multiple E-NTPDase isoforms. Some reports suggest that POM-1 also blocks several P2XRs. This may interfere with cell death and may explain their differing effects on chemotherapy-induced cell death. ENPPase and TNAP substrates are not limited to ATP and can affect other nucleotides and cyclic nucleotides, and therefore, inhibition of these enzymes may have ATP non-specific effects (46–48). Each metabolite may have different effects on cell viability, either positive or negative, and this could explain why ENPPase and TNAP increase eATP levels but do not impact cell viability.

We also showed that the addition of exogenous eATP in the absence of chemotherapy significantly reduced TNBC cell viability. Specific inhibitors of P2RX4 and P2RX7, but not a non-specific P2RX inhibitor, attenuated the effects of ATP on cell viability and the effects of E-NTPDase inhibitors on eATP levels and their positive effects on chemotherapy-induced cell death. These data show that ATP-induced cell death and E-NTPDase-inhibitor induced augmentation of chemotherapy-induced cell death are mediated through P2RX4 and P2RX7 channels. However, two observations suggest that P2RX4 and P2RX7 activation alone may not be sufficient for the loss of cell viability observed. Firstly, the addition of eATP to cells was toxic to MDA-MB 231 and Hs 578t cells but minimally affected MDA-MB 468 cells. However, upon chemotherapy treatment, an E-NTPDase inhibitor significantly increased eATP levels and augmented chemotherapy-induced cell death in all the TNBC cell lines. Secondly, the addition of eATP induced loss of TNBC cell viability at concentrations that were higher than those that were observed upon chemotherapy treatment in the presence of eATPase inhibitors. Thus, although P2RX4 and P2RX7 activation are necessary for the augmentation of chemotherapy-induced cell death by eATP, other factors may be required in parallel. For example, the NLRP3 inflammasome is one such death pathway whose activation is dependent on eATP but must also be primed by other factors such as NF-κB activation. Also, it is important to consider previous research which suggests that eATP concentrations in the immediate pericellular region may far exceed those in the bulk interstitial fluid (21). Thus, our measurements of eATP in the bulk supernatants may have underestimated pericellular eATP concentrations. In addition, ATP-induced signaling may occur in membrane demarcated intracellular organelles such as lysosomes, where ATP concentrations are independent of eATP concentrations (49).

The effects of eATP on cell viability and the effects of extracellular ATPase inhibitors on eATP levels were reversed by specific P2RX4 and P2RX7 inhibitors suggesting that these receptors are not only necessary for the accentuated cell death downstream of increased eATP but also necessary for increased eATP release. The fact that P2RX4 and P2RX7 antagonists significantly attenuated eATP release even at concentrations of paclitaxel at which cytotoxicity was similar between the treatment groups, suggests that their attenuation of eATP was not due to attenuation of cell death. Given that the P2RX4 and P2RX7 antagonists but not a non-specific P2RX blocker reversed these effects, they are likely specific to these two receptor types.

We aimed to identify clinically approved compounds that modulate eATP levels. The antiparasitic drug ivermectin is an activator of P2RX4 and P2RX7 (44, 45). We showed that consistent with this activity, ivermectin sensitized TNBC cell lines to chemotherapy. Interestingly, we also observed increased eATP release in chemotherapy-treated cells in the presence of ivermectin. This finding is consistent with our data indicating that P2RX4 and P2RX7 channels are not only necessary for ATP-induced loss of viability but also for eATP release.

Concerning expression levels, our western blot data show that P2RX4 is highly expressed in some TNBC cell lines as compared to immortal mammary epithelial cells. However, our flow cytometry data revealed significantly decreased cell surface expression of P2RX4 in the TNBC cell lines as compared to between MCF-10A cells. Previous publications suggest that the majority of P2RX4 is expressed on lysosomal membranes and that cell surface expression can be increased by stimuli that induce lysosomal exocytosis such as calcium ions (49). This may explain the different expression patterns detected by western blot and flow cytometry.

On western blot analysis of mammary cells, we detected a specific band corresponding to the full-length glycosylated form of P2RX7 in the MDA-MB 231 and MCF-10A cells and lower molecular weight bands that may represent unglycosylated forms of P2RX7 in the MDA-MB 468 and Hs 578t cells. Although expression levels may be low, given that a specific inhibitor of P2RX7 markedly attenuated the cytotoxic effects of eATP and attenuated the positive effects of E-NTPDase inhibitors on eATP levels and loss of cell viability upon chemotherapy exposure, it is possible that even low levels of expression P2RX7 may have functional consequences due to the formation of non-selective macropores in the cell membrane.

On the other hand, our flow cytometry data shows that P2RX7 is expressed at the cell surface of all the TNBC cell lines at higher levels than MCF-10A cells and in the presence of paclitaxel some TNBC cell lines expressed more P2RX7. This suggests there may be selection pressure for higher expression of P2RX7 in TNBC cell lines. Several published data support the facilitator role played by extracellular adenosine, derived from the metabolism of eATP, for the survival of cancer cells by inducing cell-autonomous effects on proliferation and cancer stem cell-like properties as well as paracrine effects on angiogenesis and immunoevasion (50–53). Additionally, this expression analysis was applied to check if expression levels of P2RX4 and P2RX7 could explain the difference in the observed effects between the MDA-MB 468 and other TNBC cell lines; the expression analysis did reveal significant differences in expression levels. This could explain differences in sensitivity of the cell lines to the eATPase inhibitors and to ivermectin.

**Figure 7:**
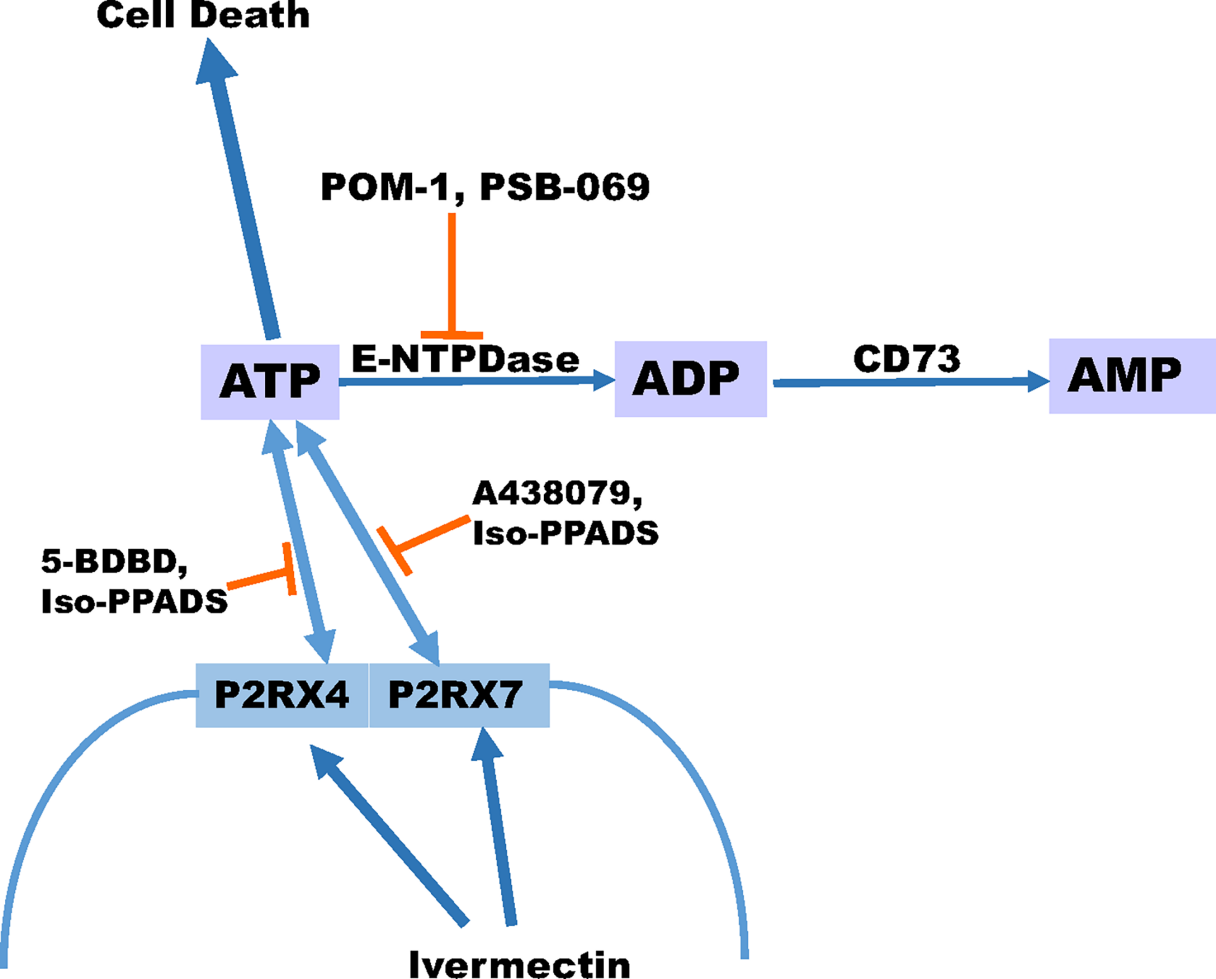
Schematic displaying our proposed model for ATP release. Our proposed model suggests that ivermectin activates P2RX4 and P2RX7 leading to the release of ATP and the more ATP that accumulates extracellular can promote cell death especially in the presence of paclitaxel. In addition, the breakdown of ATP can be prevented in the presence of E-NTPDase inhibitors POM-1 or PSB 069. However, the release of ATP can be prevented in the presence of P2RX4 inhibitors 5-BDBD or Iso-PPADS or P2RX7 inhibitors A438079 or Iso-PPADS.

In summary, our data indicate that eATP is toxic to several TNBC cell lines and P2RX4 and P2RX7 purinergic channels are necessary for this effect. Chemotherapy exposure induces the release of ATP from TNBC cell lines and inhibitors of eATP metabolism augment chemotherapy-induced loss of TNBC cell viability, and these effects are reversed by specific inhibitors of P2RX4 and P2RX7, suggesting that both eATP release and eATP induced loss of cell viability are mediated by these channels. A heterocyclic-sulphonate inhibitor of multiple E-NTPDases, PSB 069, was most effective at accentuating chemotherapy-induced cell death. A P2RX4 and P2RX7 activator, ivermectin, also accentuated chemotherapy-induced increases in eATP and loss of TNBC cell viability **(** **Figure 7****)**. Although only E-NTPDase inhibitors consistently increased chemotherapy-induced loss of cell viability, all the different classes of extracellular ATPase inhibitors increased eATP levels in the setting of chemotherapy exposure. Thus, to maximally augment eATP levels and reduce adenosine in the tumor microenvironment, inhibitors that have broad inhibitory effects on multiple classes of extracellular ATPases may be necessary. This is in contrast to current monoclonal antibody-based strategies that narrowly focus on E-NTPDase1/CD39 (54). Our future goals are to examine the effects of eATPase inhibition and P2RX4 and P2RX7 activation on TNBC models *in vivo* in the context of an intact tumor microenvironment and functional immune system. These preclinical experiments may lead to therapeutic strategies for TNBC that modulate purinergic signaling in the tumor microenvironment.

## Data Availability Statement

The raw data supporting the conclusions of this article will be made available by the authors, without undue reservation.

## Author Contributions

All authors contributed to review and analysis. JMM performed a majority of the assays with NW executing the RNA analysis and JD carrying out the Western blot analysis. JM and MAC conceived of and designed the experiments, reviewed the data, authored and edited the manuscript. All authors contributed to the article and approved the submitted version.

## Funding

Research reported in this publication was supported by The Ohio State University Comprehensive Cancer Center. Institutions that provided funding support had no role in the design or conduct of this study or the preparation of the manuscript. This publication was also supported, in part, by the National Center for Advancing Translational Sciences of the National Institutes of Health under Grant Numbers KL2TR002734. The content is solely the responsibility of the authors and does not necessarily represent the official views of the National Institutes of Health.

## Conflict of Interest

The authors declare that the research was conducted in the absence of any commercial or financial relationships that could be construed as a potential conflict of interest.

## Publisher’s Note

All claims expressed in this article are solely those of the authors and do not necessarily represent those of their affiliated organizations, or those of the publisher, the editors and the reviewers. Any product that may be evaluated in this article, or claim that may be made by its manufacturer, is not guaranteed or endorsed by the publisher.
