# Supplementary material for: Augmentation of Extracellular ATP Synergizes With Chemotherapy in Triple Negative Breast Cancer
Source: Front Oncol. 2022 Apr 20;12:855032. doi: 10.3389/fonc.2022.855032 (PMC9065442; doi:10.3389/fonc.2022.855032)
Supplement: Supplementary file 1 [file DataSheet_1.pdf]

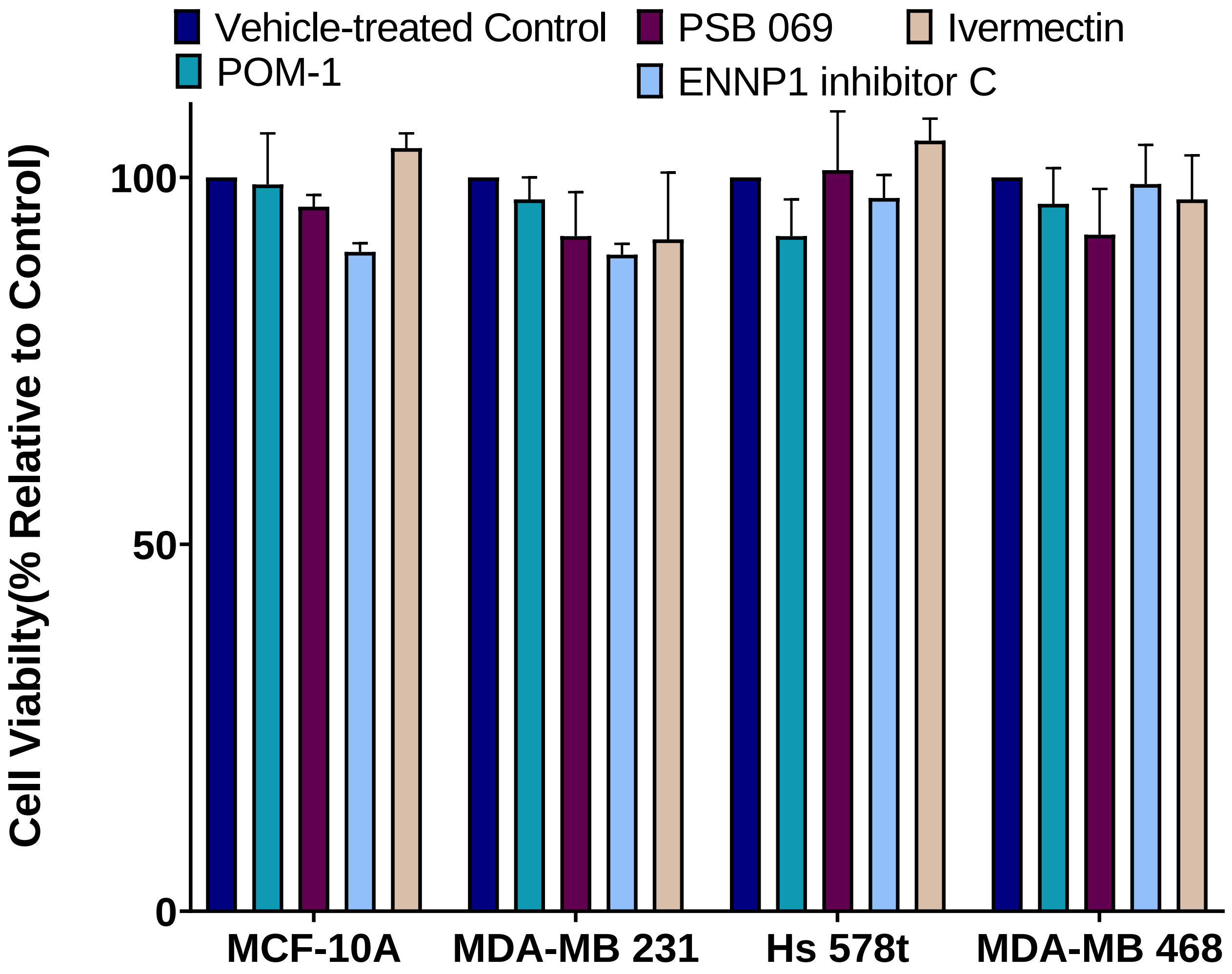

Figure S1: Cell viability of eATPase inhibitors. TNBC and MCF-10A cell lines were treated with POM-1 (E-NTPDase inhibitor, 10 µmol/L), PSB 069 (E-NTPDase inhibitor, 10 µmol/L), ENNP1 inhibitor C (ENPP1 inhibitor, 10 µmol/L) or vehicle addition for six hours, and cell viability was measured using the PrestoBlue HS assay. Standard deviation was calculated from three independent experiments performed in triplicate.

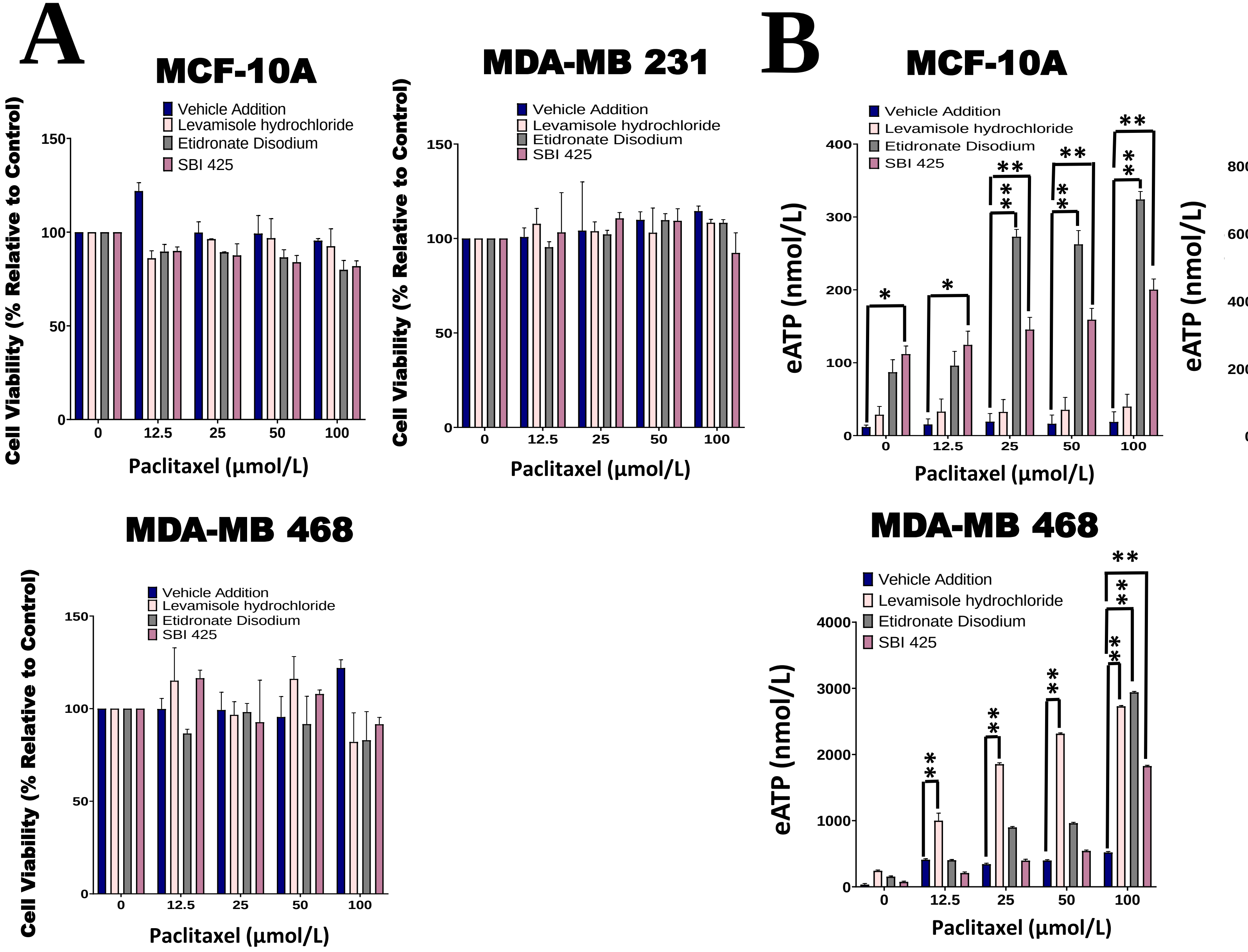

Figure S2: Comparing eATP release from paclitaxel-treated cells in the presence of phosphatase inhibitors or vehicle addition. (A) TNBC cell lines and MCF-10A cells were treated for six hours with phosphatase inhibitors levamisole hydrochloride (tissue non-specific alkaline phosphatase inhibitor, 50 µmol/L), SBI 425 (tissue non-specific alkaline phosphatase inhibitor, 10 µmol/L), etidronate disodium (protein tyrosine phosphatase inhibitor, 50 µmol/L) or vehicle addition, and cell viability was measured with PrestoBlue HS. Standard deviation was calculated from three independent experiments performed in triplicate. (**B)** eATP concentrations were measured in the supernatants of paclitaxel-treated TNBC cell lines and MCF-10A cells in the presence of the phosphatase inhibitors levamisole, SBI 425, etidronate sodium, or vehicle addition with paclitaxel. One way ANOVA was performed to ascertain significance. * represents $p<0.05$ and ** represents $p<0.01$ when comparing vehicle addition to levamisole, SBI 425 or etidronate sodium.

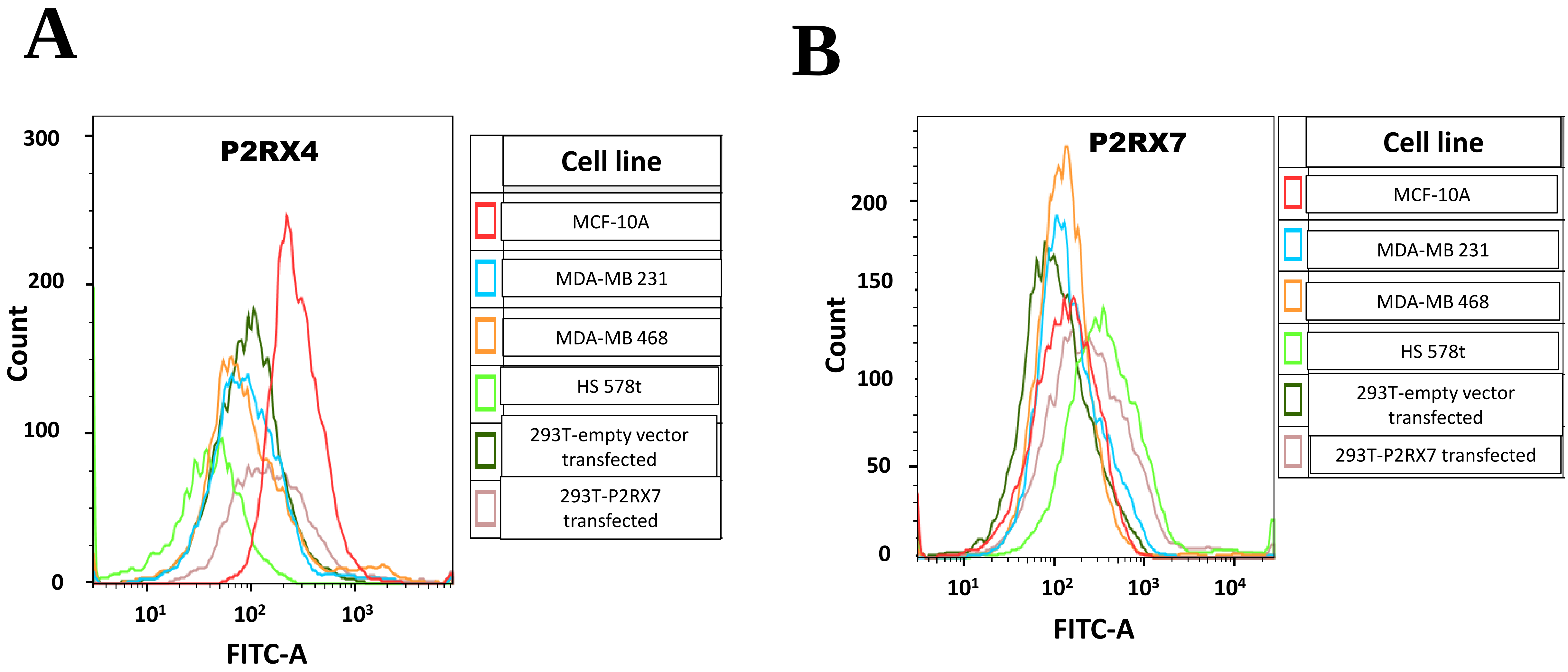

Figure S3: Cell surface expression analysis of P2RX4 and P2RX7. Histograms displaying the different extracellular (**A)** P2RX4 and (**B)** P2RX7 expressions in TNBC cell lines, MCF-10A cells, and HEK 293T cells transfected with P2RX4 or P2RX7 as positive controls.

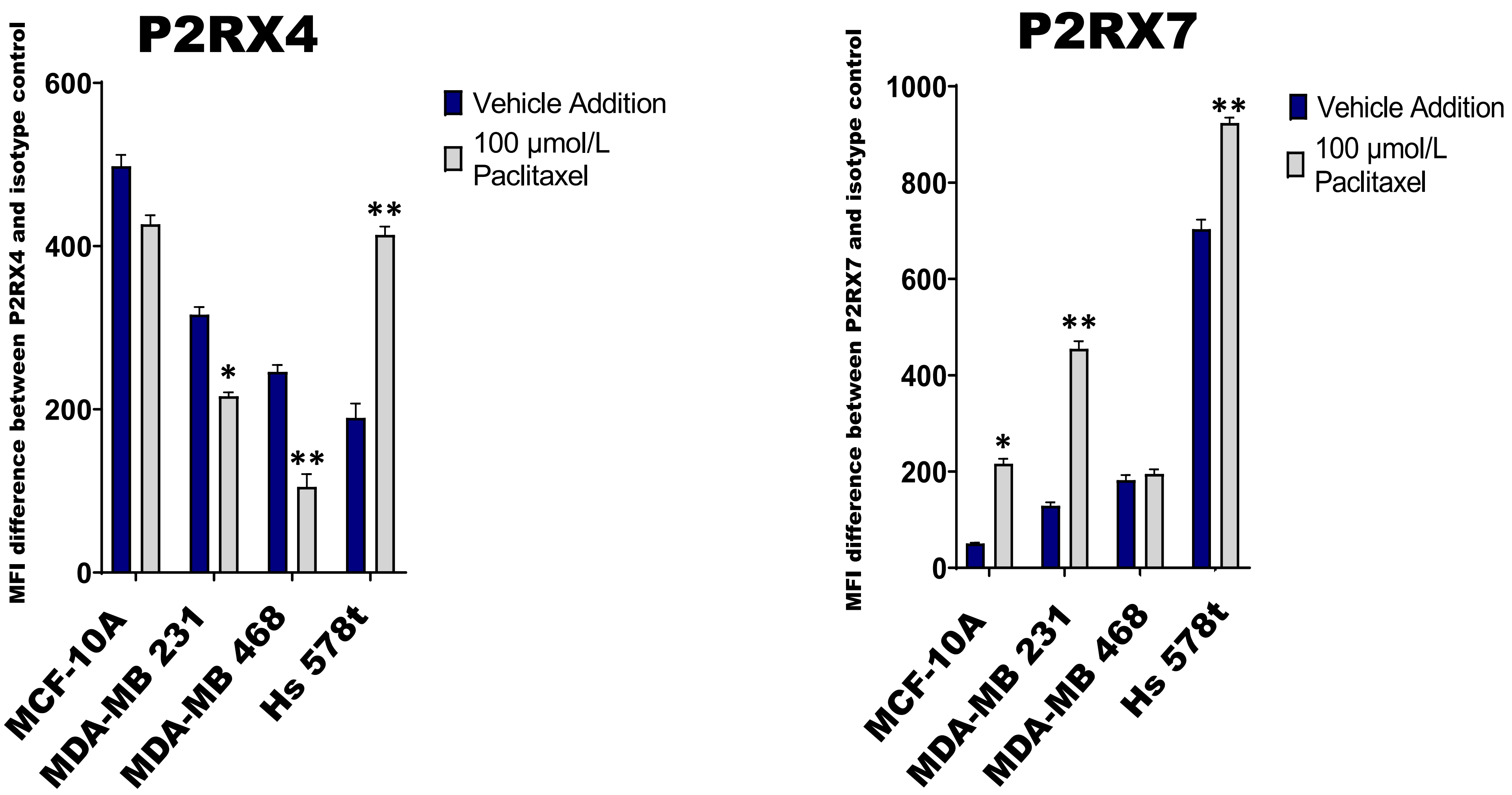

Figure S4: Paclitaxel's impact on the cell surface expression of P2RX4 and P2RX7. The calculated difference in mean fluorescence intensity (MFI) values between TNBC cell lines and MCF-10A cells in the absence of presence of 100 µmol/L paclitaxel stained with **(A)** P2RX4 or **(B)** P2RX7 specific antibody and the isotype control for the different cell lines examined. Student's t-test was performed to ascertain significance. * represents $p<0.05$ and ** represents $p<0.01$ relative to MFI difference in MCF-10A cells.
